# An Enhanced Positional Error Compensation Method for Rock Drilling Robots Based on LightGBM and RBFN

**DOI:** 10.3389/fnbot.2022.883816

**Published:** 2022-05-13

**Authors:** Xuanyi Zhou, Wenyu Bai, Jilin He, Ju Dai, Peng Liu, Yuming Zhao, Guanjun Bao

**Affiliations:** ^1^Key Laboratory of Special Purpose Equipment and Advanced Processing Technology, Zhejiang University of Technology, Hangzhou, China; ^2^Zhejiang Jinbang Sports Equipment Co. Ltd., Zhejiang, China; ^3^State Key Laboratory of High Performance Complex Manufacturing, Central South University, Changsha, China; ^4^Sunward Intelligent Equipment Company, Ltd., Changsha, China; ^5^Department of Precision Instruments, Tsinghua University, Beijing, China

**Keywords:** rock drilling robot, error compensation, parameter identification, parallel differential evolution algorithm, RBFN

## Abstract

Rock drilling robots are able to greatly reduce labor intensity and improve efficiency and quality in tunnel construction. However, due to the characteristics of the heavy load, large span, and multi-joints of the robot manipulator, the errors are diverse and non-linear, which pose challenges to the intelligent and high-precision control of the robot manipulator. In order to enhance the control accuracy, a hybrid positional error compensation method based on Radial Basis Function Network (RBFN) and Light Gradient Boosting Decision Tree (LightGBM) is proposed for the rock drilling robot. Firstly, the kinematics model of the robotic manipulator is established by applying MDH. Then a parallel difference algorithm is designed to modify the kinematics parameters to compensate for the geometric error. Afterward, non-geometric errors are analyzed and compensated by applying RBFN and lightGBM including features and kinematics model. Finally, the experiments of the error compensation by combing combining the geometric and non-geometric errors verify the performance of the proposed method.

## 1. Introduction

The tunnel construction machine has been playing an increasingly important role in the development of the modern economy due to the continuous expansions of anthropogenic activity. Moreover, the drilling and blasting method is the most practical and effective method for tunnel engineering (Ocak and Bilgin, 2010). Rock drilling robots are one of an essential piece of tunnel equipment used for drilling and blasting methods. They have been widely applied in the tunnel construction due to its their advantages for reducing labor intensity. Robotic drilling can effectively prevent overcutting and undercutting to improve the efficiency and quality of the tunnel. However, due to the characteristics of the heavy load, large span, and multi-joints of the robotic manipulator, the errors of the rock drilling robot are diverse and non-linear, which pose challenges to the intelligent and high-precision control of the robotic manipulator.

Position accuracy is an important parameter for the robot (Chen D. et al., 2018; Qi et al., 2021). High positioning accuracy is the a guarantee for complex robotic tasks. Especially for industrial robots and medical robots, the error compensation method is important for efficiency and safety. Now robotic surgery is gradually replacing manual surgery because of its stability and convenience, and high precision is the most important guarantee element needed for safety (Su et al., 2018, 2021). These electrical robots for the industry can achieve high accuracy of up to a millimeter level with a high-precision motor (Su et al., 2022). However, the accuracy of hydraulic robots is limited by the underlying rigid actuation mechanisms of the hydraulic actuators. With the improvement of accuracy, the price of hydraulic components has also increased substantially. Therefore, it is necessary to use error compensation methods for the hydraulic robot utilizing conventional hydraulic actuators.

In general, considering the characteristics of nonlinear and multi-coupling, the robotic error is very complex. With the advancement of computer technology, numerous non-linear fitting algorithms are proposed, such as Artificial Neural Networks (ANN) (Zhang et al., 2021), Radial Basis Function Network (RBFN) (Park and Sandberg, 1991), and extreme learning machine (ELM) (Huang et al., 2004), etc. Huang proposed a method to assess the critical parameters for short-term wind generation forecasting by ANN (Sewdien et al., 2020). Chen D. et al. (2019) proposed a positional error compensation combing RBFN and error similarity. RBFN is used to estimate the error of the target positions. Yuan et al. (2018) demonstrated that a trained ELM method could guarantee high position accuracy on the drilling robot and reduce the working time and workload. Neural networks have a very wide application. However, it needs many super-parameters to set and takes too long to train the process.

Decision Tree (DT) is a supervised learning method that is widely used to predict models based on a tree structure. DT has higher decision-making efficiency in comparison with other machine learning methods. In fact, many researchers have improved DT models, and some more optimized algorithms have been proposed, such as Random Forest (RF) (Breiman, 2001; Svetnik et al., 2003), Gradient Boosting Decision Tree (GBDT) (Ke et al., 2017), eXtreme Gradient Boosting (XGBoost) (Naghibi et al., 2020), and so on. The liquid crystalline behaviors are successfully predicted using RF in Chen C. H. et al. (2018). Yao et al. (2019) proposed a method for predicting line loss rate in a low voltage distribution network based on GBDT method. By analyzing and verifying the data, the GBDT method is accurate and effective. Light-GBM is also an improved algorithm based on GBDT, which was firstly proposed by Microsoft Research Asia in 2016 (Chen P. et al., 2019). Zhou et al. (2020) proposed a hybrid reservoir permeability prediction method based on Light-GBM. The results show that the method has excellent prediction ability. Compared with the DT algorithm, Light-GBM can significantly promote the training speed without decreasing the accuracy and also occupy less memory during the training process.

There are two types of positional errors in robotic control. The first error is a geometric error, which is generated by the mechanical manufacturing error and assembling error of the robot. To reduce the geometric errors, a parallel difference algorithm is designed in this article to modify the kinematics parameters (Praveen and Denis, 2018; Zhu et al., 2020). The second error is generated by environmental factors, such as temperature and gravity, etc., that have nothing to do with the robot itself, which are defined as non-geometric errors (Zeng et al., 2017; Jiang et al., 2021). These non-geometric errors are usually difficult to analyze accurately because of their non-linear characteristics. Specifically for the manipulators as long as 15 meters, the accuracy is affected by many factors such as elastic deflection and hydraulic components. Nevertheless, Atlas Copco, the world-leading rock drilling robot corporation, can achieve an accuracy of up to 100 mm (Molfino et al., 2008).

In order to enhance the control accuracy, a hybrid positional error compensation method based on Radial Basis Function Network (RBFN) and Light Gradient Boosting Decision Tree (LightGBM) is proposed for the rock drilling robot. Firstly, the kinematics model of the robotic manipulator is established by applying MDH. Then a parallel difference algorithm is designed to modify the kinematics parameters to compensate for the geometric error. Afterward, non-geometric errors are analyzed and compensated by applying RBFN and lightGBM, including features and the kinematics model. Finally, the experiments of the error compensation by combing combining the geometric and non-geometric errors verify the performance of the proposed method.

The structure of the article is divided into five parts. The first part is the introduction. The second part is the kinematic model of the rock drilling robot. According to the structural characteristics of the manipulator, the MDH method is applied to establish the kinematics model of the manipulator. The third part is methodology. By analyzing the parameters of the geometric error compensation model, a parallel difference error compensation algorithm is designed to identify the parameters. Moreover, RBFN and lightGBM are applied to compensate for the non-geometric errors. The fourth part is experimental validation and results. The error compensation experiment is carried out, and the effectiveness of the proposed method is verified. The fifth part is the discussion and summary, which discusses the advantages of the error compensation method.

## 2. Kinematic Model of the Rock Drilling Robot

### 2.1. Mechanical Structure of the Rock Drilling Robot

**Figure 1:**
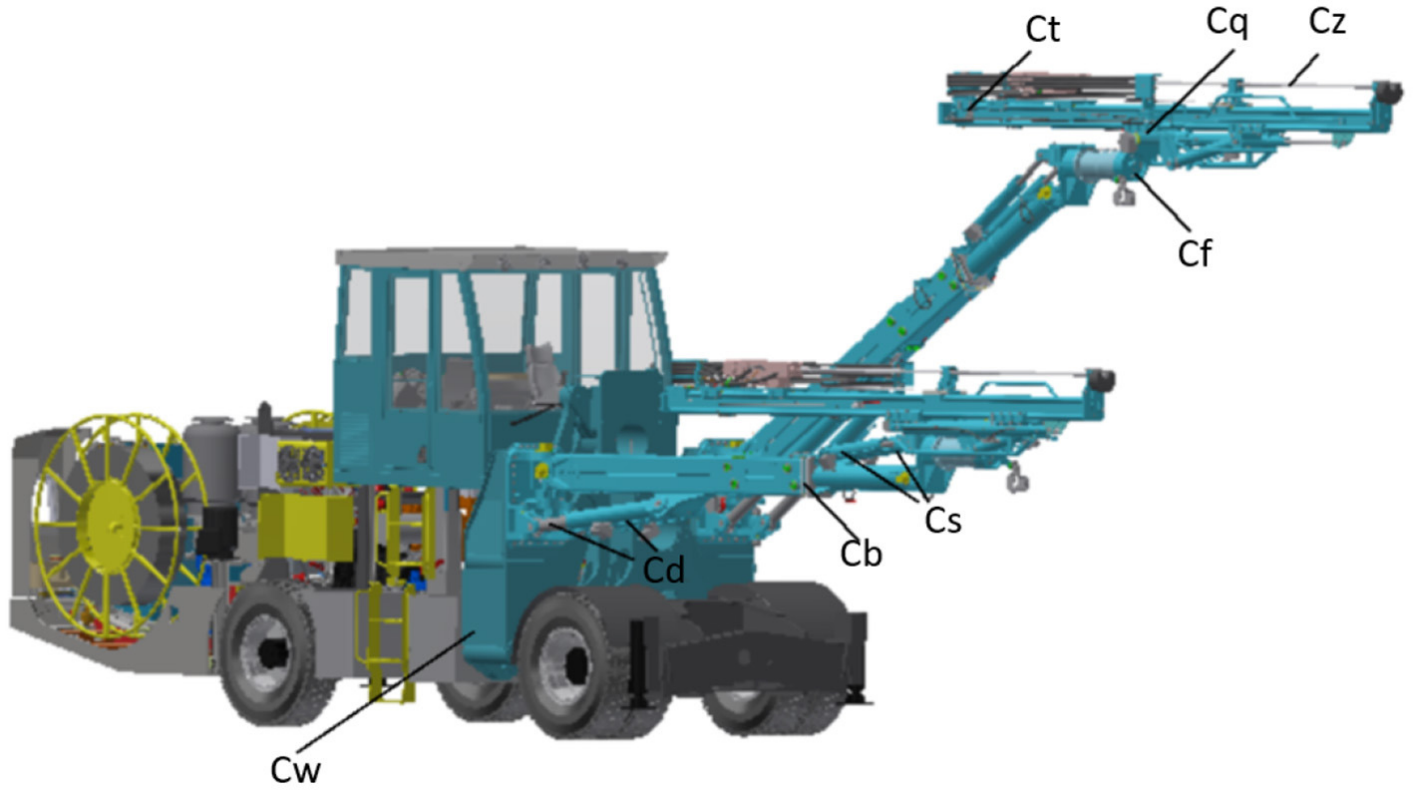
The structure of a rock drilling robot, *C*_*w*_, wingspan cylinder; *C*_*d*_, large triangle left and right manipulator oil cylinder; *C*_*b*_, telescopic oil cylinder; *C*_*s*_, small triangle left and right pitching oil cylinder; *C*_*f*_, turnover hydraulic motor; *C*_*q*_, tilt oil cylinder; *C*_*t*_, compensation oil cylinder; *C*_*z*_, drill rod oil cylinder.

The rock drilling robot is mainly composed of the wingspan platform, left and right drilling manipulator, as shown in Figure 1. The wingspan platform *C*_*w*_ can be raised to enhance the work area. The drilling manipulator, which is mounted at the end of the wingspan, is the main working device of the rock drilling robot. The drilling manipulator has three moving joints and six rotating joints. The left and right manipulator oil cylinders *C*_*d*_ are placed at the front end of the manipulator to form a double triangle structure. At the same time, the left and right pitching cylinders *C*_*s*_ form a small triangle structure and are connected in series with the manipulator front support cylinder *C*_*d*_ to realize parallel linkage at the rear end of the manipulator. The extension of the manipulator telescopic cylinder *C*_*b*_ determines the longitudinal working distance of the manipulator. The hydraulic motor *C*_*f*_ of the flip joint is used to control the propeller around the axis Linear lateral rotation, with thruster tilt cylinder *C*_*q*_ to achieve different drilling angles. Compensation cylinder *C*_*t*_ pushes the bracket to move forward and backward to realize the fine adjustment of the position and posture. Each of the joints is actuated by hydraulic cylinders, which effectively serve as velocity sources.

### 2.2. Forward Kinematics Based on MDH Model

DH method is a standard method to realize the transformation between joint variables and Cartesian coordinates and learn the kinematics modeling of the robot. However, when the joint axes between adjacent links are close to parallel, the position of the actual normal will deviate significantly from the theoretical normal due to the slight deviation of parallelism, which will affect the kinematic calculation.

Therefore, the MDH method is adopted to describe the conversion relationship of parallel joint axis systems. Its characteristic role is to add the rotation change Rot (*y*_*i*_, β_*i*_) around the y axis on the original DH model and set the initial value of β_*i*_ of adjacent joints to 0.

**Figure 2:**
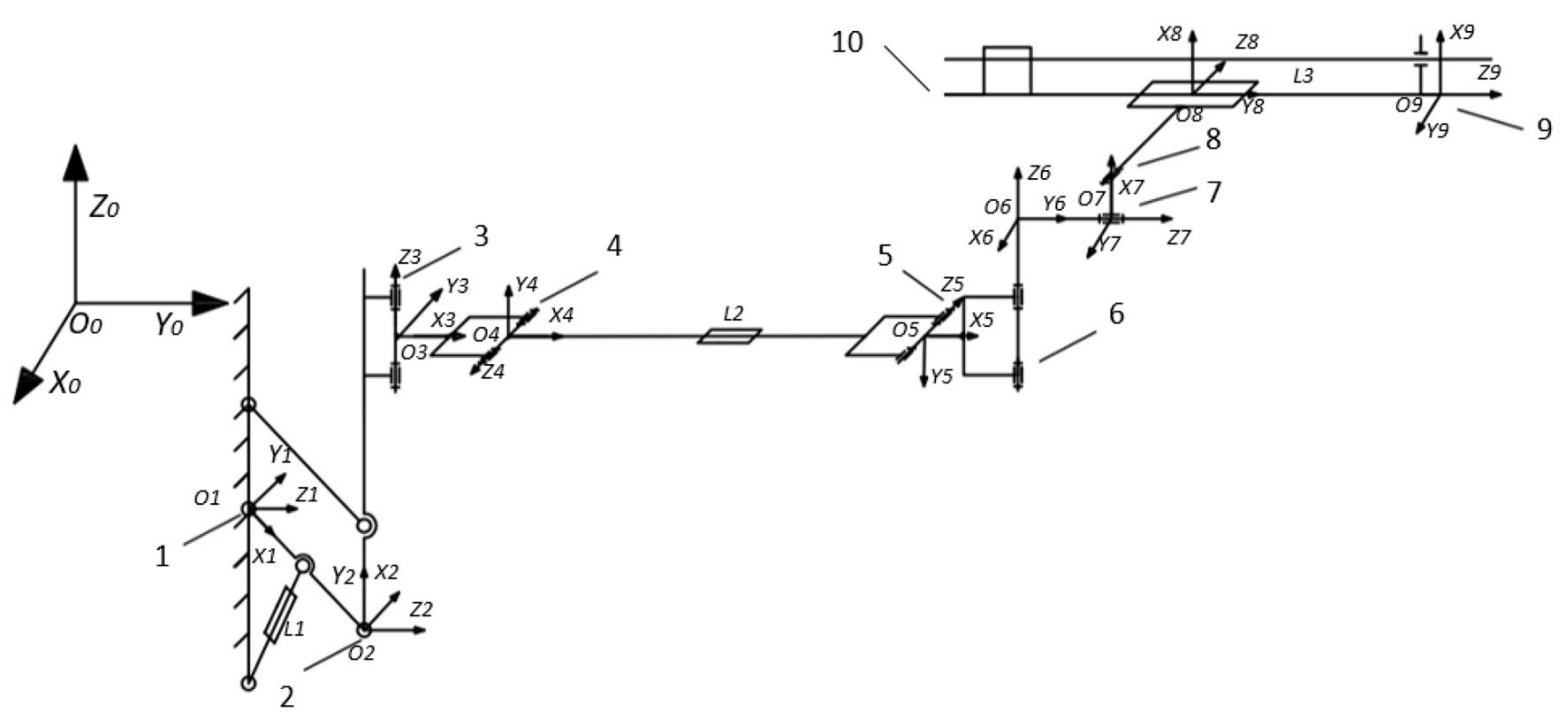
Kinematics of rock drilling manipulator. Joint 1, wingspan rotation joint; Joint 2, wingspan parallel joint; Joint 3, left and right rotation joint of big triangle; Joint 4, pitch joint of big triangle; Joint 5, pitch joint of small triangle; Joint 6, left and right rotation joint of small triangle; Joint 7, turning joint; Joint 8, tilt joint; Joint 9, front end of compensation joint; Joint 10, end of compensation joint; L1, span support oil cylinder; L2, telescopic oil cylinder of large manipulator; L3, compensation propulsion cylinder.

According to the structural characteristics of the rock drilling manipulator, the coordinate system transformation between links is designed, and the kinematic model of the manipulator is established, as shown in Figure 2.

### 2.3. Forward Kinematics Model With MDH

When the coordinate systems of the manipulator 1, 2, 4, and 5 are parallel, the forward kinematics model of the rock drilling robot is established according to MDH method as shown in Table 1, and the homogeneous transformation matrix $T_{i}^{i-1}$ between adjacent link coordinate systems is obtained, as shown in Equation (1):


(1) $$\begin{array}{l} \begin{array}{c} T_{i}^{i-1}\left(\alpha_{i-1},a_{i-1},d_{i},\theta_{i},\beta_{i}\right)\\ =\mathrm{Trans}\left(x,a_{i-1}\right)\mathrm{Rot}\left(x,\alpha_{i-1}\right)\\ \mathrm{Trans}\left(z,d_{i}\right)\mathrm{Rot}\left(z,\theta_{i}\right)\mathrm{Rot}\left(y,\beta_{i}\right)\\ =\left[\begin{array}{cccc} m_{11} & m_{12} & m_{13} & m_{14}\\ m_{21} & m_{22} & m_{23} & m_{24}\\ m_{31} & m_{32} & m_{33} & m_{34}\\ 0 & 0 & 0 & 1 \end{array}\right] \end{array} \end{array}$$

where *m*_11_ = *cθ*_*i*_*cβ*_*i*_, *m*_12_ = −*sθ*_*i*_, *m*_13_ = *cθ*_*i*_*sβ*_*i*_, *m*_14_ = *a*_*i*−1_, *m*_21_ = *sθ*_*i*_*cα*_*i*−1_*cβ*_*i*_ + *sα*_*i*−1_*sβ*_*i*_, *m*_22_ = *cθ*_*i*_*cα*_*i*−1_, *m*_23_ = *sθ*_*i*_*cα*_*i*−1_*sβ*_*i*_−*sα*_*i*−1_*cβ*_*i*_, *m*_24_ = −*d*_*i*_*sα*_*i*−1_, *m*_31_ = *sθ*_*i*_*sα*_*i*−1_*cβ*_*i*_−*cα*_*i*−1_*sβ*_*i*_, *m*_32_ = *cθ*_*i*_*sα*_*i*−1_, *m*_33_ = *sθ*_*i*_*sα*_*i*−1_*sβ*_*i*_ + *cα*_*i*−1_*cβ*_*i*_, *m*_34_ = *d*_*i*_*cα*_*i*−1_. *s* presents the sine function while *c* is the cosine function.

**Table 1:** DH parameter of the rock drilling robot.

**i**	**Variable**	**Variable range**	**α_*i*−1_/(°)**	***a*_*i*−1_/mm**	***d*_*i*_/mm**	**θ_*i*_/(°)**	**β_*i*_/(°)**
1	θ_1_	−15 to 60°	90°	200	250	θ_1_	0
2	θ_2_	15 to 60°	0	1,382	0	−θ_1_	0
3	θ_3_	30 to 150°	−90°	0	2,500	θ_3_	0
4	θ_4_	−15 to 60°	−90°	0	θ_4_	0	0
5	*L*_2_,θ_5_	0~2,168 mm, −30 to 30°	0	5,000+*L*_2_	0	θ_5_	0
6	θ_6_	−75 to −125°	90°	145	0	θ_6_	0
7	θ_7_	−25 to −175°	−90°	0	900	θ_7_	0
8	θ_8_	15 to −20°	90°	240	580	θ_8_	0
9	*L*_3_	0 to 1,778 mm	−90°	0	2,230+*L*_3_	0	0

When the coordinate systems of two adjacent joints are not parallel, β_*i*_ = 0, and the second transformation $T_{i}^{i-1}$ between the links of two adjacent non-parallel joints is defined in Equation (2):


(2) $$\begin{array}{l} \begin{array}{c} T_{i}^{i-1}\left(\alpha_{i-1},a_{i-1},d_{i},\theta_{i}\right)\\ \;=\mathrm{Rot}\left(x,\alpha_{i-1}\right)\mathrm{Trans}\left(x,a_{i-1}\right)\mathrm{Rot}\left(z,\theta_{i}\right)\mathrm{Trans}\left(z,d_{i}\right)\\ \;=\left[\begin{array}{cccc} c\theta_{i} & -s\theta_{i} & 0 & a_{i-1}\\ s\theta_{i}c\alpha_{i-1} & c\theta_{i}c\alpha_{i-1} & -s\alpha_{i-1} & -d_{i}s\alpha_{i-1}\\ s\theta_{i}s\alpha_{i-1} & c\theta_{i}s\alpha_{i-1} & c\alpha_{i-1} & d_{i}c\alpha_{i-1}\\ 0 & 0 & 0 & 1 \end{array}\right] \end{array} \end{array}$$

Substituting the parameters of each joint into the homogeneous transformation matrix, the transformation matrix of the robot manipulator end actuator relative to the base is obtained, as shown in Equation (3):


(3) $$\begin{array}{l} \begin{array}{c} T_{10}^{0}=\left[\begin{array}{cccc} n_{x} & o_{x} & a_{x} & P_{x}\\ n_{y} & o_{y} & a_{y} & P_{y}\\ n_{z} & o_{z} & a_{z} & P_{z}\\ 0 & 0 & 0 & 1 \end{array}\right]=\left[\begin{array}{cc} R & P\\ 0 & 1 \end{array}\right] \end{array} \end{array}$$

## 3. Methodology

### 3.1. Parameter Identification of Geometric Error Compensation Model

The geometric error mainly refers to the error between the ideal kinematic model parameters and the parameters of the actual MDH modeling (Khan and Chen, 2011; Cui et al., 2012). Therefore, the geometric errors are composed of measurement errors of length and angles during modeling, the calibration errors of the sensor, the manufacturing errors, and assembly errors. For positive kinematics parameters, *a*_*i*_ represents the distance between the joint axes of two adjacent joints, and the main error caused by *a*_*i*_ is the actual machining error. The symbol α_*i*_ represents the relative rotation angle of the two connected joints, and its main error comes from the coaxiality error in the assembly. *d*_*i*_ represents the relative distance between the two joints on the common joint axis, which is mainly derived from the measurement error during modeling. θ_*i*_ represents the rotation angle of two adjacent joints around the common axis; the error mainly comes from the observation error of the sensors.

The key role of the MDH method is to determine the axis of each joint. Therefore, by tracking and recording the trajectory of each joint's movement, the actual joint axis position of the robot can be established in the MDH model. The kinematic parameters of the manipulator are defined by error computation or estimation applying the posture measurement and position data of the end effector. In this article, the kinematic parameters are estimated by applying a differential evolution algorithm. The identified kinematics error parameters Δα, Δ*a*, Δ*d*, Δθ, Δβ are defined as: Kinematic parameters are estimated applying differential evolution algorithm. The identified kinematics error parameters Δα, Δ*a*, Δ*d*, Δθ, Δβ are defined as:


(4) $$\begin{array}{l} F\left(\Delta \alpha ,\Delta a,\Delta d,\Delta \theta ,\Delta \beta \right)=\sum \left(e_{d}^{2}+e_{\delta }^{2}\right) \end{array}$$

where *e*_*d*_ denotes the error of the end position, which indicates the distance deviation between the predicted position of the model and the actual position. The symbol *e*_δ_ is the error of the end attitude, which indicates the predicted rotation angle deviation between the model and the actual attitude on the x, y, and z axes. The observation prisms “r” and “s” are set at the front and end of the manipulator's compensation cylinder. The displacement from the deflection deformation and other nonlinear factors are ignored in the geometric compensation. Then the attitude error at the end of the robotic manipulator $\left[\begin{array}{cc} e_{d}^{r} & e_{\delta }^{r} \end{array}\right]$ can be obtained from the position error of the two observation $\left[\begin{array}{cc} e_{d}^{r} & e_{d}^{s} \end{array}\right]$. The geometric error model can be expressed as:


(5) $$F(\Delta \alpha ,\Delta a,\Delta d,\Delta \theta ,\Delta \beta )=\sum \left(e_{d}^{r}{}^{2}+e_{d}^{s}{}^{2}\right)$$

### 3.2. Parallel Differential Evolution

The differential evolution algorithm is a heuristic algorithm that can be applied to solve the optimization problem through competition and cross-mutation. In order to identify the parameters of the rock drilling robot, the common differential evolution algorithm is proposed as the following five steps:

**Step 1** Initialization

The initial population is generated by randomly sampling the feasible search space defined by joint bounds, as shown in Equation (6):


(6) $$\begin{array}{l} x_{ij}\left(0\right)=L_{j}+{\mathrm{rand}}_{ij}\left(0,1\right)\left(U_{j}-L_{j}\right) \end{array}$$

Let *n* be the individual dimension, expressed as the value of the first individual of the generation population in the dimension, and the individual randomly generates an initial value that meets the constraints in each dimension:


(7) $$\begin{array}{l} x_{ij}\left(0\right)=L_{j}+{\mathrm{rand}}_{ij}\left(0,1\right)\left(U_{j}-L_{j}\right) \end{array}$$

In Equation (6), *U*_*j*_ and *L*_*j*_ represent the upper and lower bounds of the individual on the dimension and *rand*_*ij*_(0, 1) represents a random number between [0, 1] that obeys a uniform distribution.

**Step 2** Mutation

There are multiple solutions for the parameter identification of the rock drilling robot error model, the robust DE/rand/1/bin evolution model is used to preserve the diversity of the search population (Mlakar et al., 2015). The specific operation is to select an individual with the highest fitness from the current population, randomly select three different individuals, and scale the difference between the two vectors and add it to another individual vector to obtain a new variable Individual (Pavelski et al., 2016):


(8) $$\begin{array}{l} v_{i}\left(t\right)=x_{p1}\left(t\right)+F\left(x_{p2}-x_{p3}\right) \end{array}$$

where F is the scaling factor of DE, the value range is [0, 1].

**Step 3** Boundary constraint

The individual vector after the mutation operation may exceed the boundary of the search solution space, and the infeasible solution is converted to generate a new vector (Tian et al., 2020):


(9) $$v_{ij}(t)=\{\begin{array}{l} \min\left\{U_{j},2L_{j}-v_{ij}(t)\right\},\text{if }v_{ij}<L_{j}\\ \max\left\{L_{j},2U_{j}-v_{ij}(t)\right\},\text{if }v_{ij}>U_{j} \end{array}$$

**Step 4** Crossover

The differential evolution algorithm crosses the reference vector and mutation vector to increase the diversity of the next generation population. The specific operations are as follows:


(10) $$h_{ij}(t+1)=\{\begin{array}{l} v_{ij}(t+1),{\text{ if rand }}_{j}[0,1]<Cr\text{ or }j=j_{\text{rand}}\\ x_{ij}(t),\text{ Otherwise } \end{array}$$

Where: rand[0,1] represents the uniformly distributed random number on the jth dimension, C_*r*_ represents the crossover probability in the range of [0,1], j_rand_ represents a random number in 1,2,…,n.

**Step 5** Selection

Based on the greedy selection mechanism, it compares the fitness of the mutated and cross-generated individual with the original individual and retains the highly adaptive individual as the next-generation population individual. The specific expression is as follows:


(11) $$X_{i}(t+1)=\{\begin{array}{c} H_{i}(t+1),\text{ if }f\left(H_{i}(t+1)\right)>f\left(X_{i}(t)\right)\\ X_{i}(t),\text{ if }f\left(H_{i}(t+1)\right)<f\left(X_{i}(t)\right) \end{array}$$

where *f* is the fitness function.

**Figure 3:**
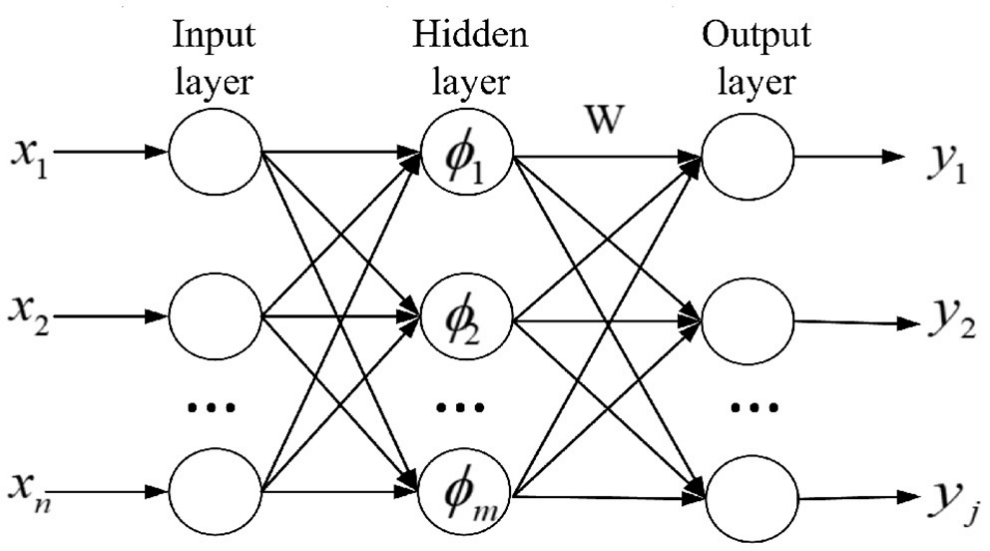
The structure of the RBFN network.

**Figure 4:**
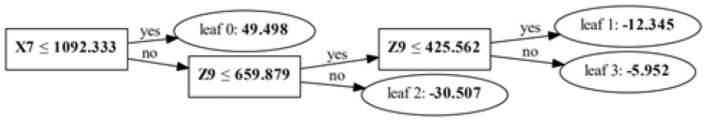
One of the regression tree of lightGBM.

**Figure 5:**
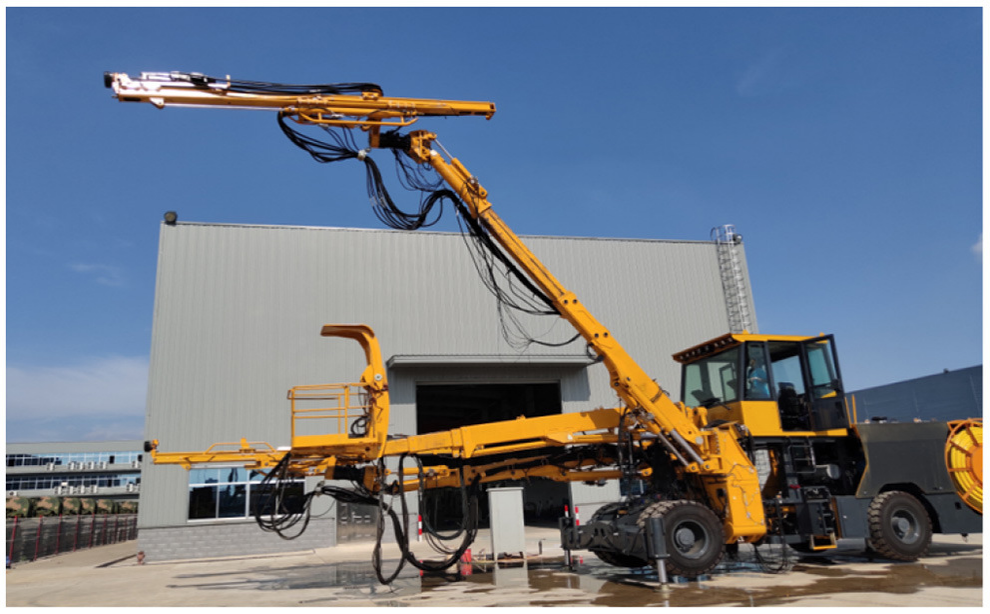
SWDT82 rock drilling robot.

**Figure 6:**
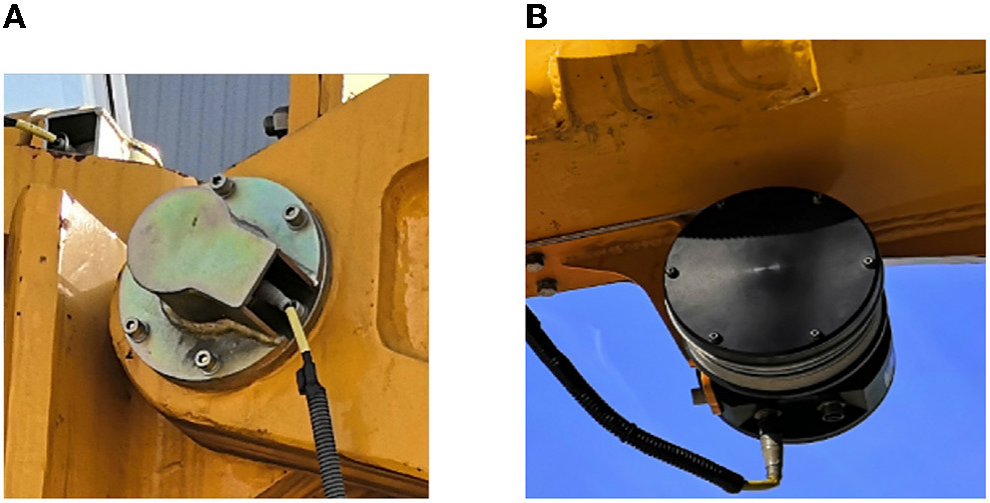
The sensors applied in the rock drilling robot, **(A)** a joint angle sensor, **(B)** is a length transducer.

Since the rock drilling robot has three moving joints and six rotating joints, the dimension numbers of the error compensation model is 38. The high-dimensional solution requires a high number of populations and evolutionary algebra, which poses challenges to computer performance, algorithm model convergence, and computing speed. In order to accelerate the model convergence and increase the calculation speed under the same computing power input, a parallel structure of the differential evolution algorithm is proposed. The computing performance of the CPU can be enhanced by applying a parallel differential evolution algorithm to calculate independent individual populations. Specifically, due to the independence of the sub-populations, multiple processes can be allocated through different computing cores of the CPU to calculate the fitness of multiple sub-populations parallel. The structure of the parallel differential evolution algorithm is shown in Figures 2–**6**. Set the kinematic parameter error compensation vector Δα, Δ*a*, Δ*d*, Δθ, Δβ to be the population individual in the differential evolution algorithm. The sum of squared errors between the trajectory of the end of the robotic manipulator and the observation trajectory in the population is *F*.

### 3.3. Non-geometric Error Analysis

Due to the heavy load, large span, redundant joints, and hydraulic character of the rock drilling robot, the error shows a high degree of non-linearity. After geometric error compensation is performed on the positive kinematics model, there are still errors due to the non-linearity. The non-geometric errors are composed of regular errors and irregular errors. Regular errors refer to predictable errors, including elastic deformation due to changes in load, frictional force direction, structural changes, and the cumulative error caused by the transducer. The irregular errors mainly come from observation errors and system errors. The observation errors are related to the dynamic position capture accuracy of the total station, the observation elevation angle, and the measurement accuracy of the optical sensor; the system errors mainly come from the command error of the encoder and the signal transmission communication delay, vibration errors caused by kinetic energy changes, etc. Regular non-geometric errors are related to the time and space of the robot arm movement. Take the compensation joint L3 as an example. Moreover, changes in the relative speed and direction of joint motion affect the magnitude and direction of frictional resistance, and thereby the position error is changed.

Features are important conditions that affects the machine learning models. Important features are extracted from the original data for the use of algorithms and models (Yu et al., 2017; Wen et al., 2021). Different algorithms have different feature selections during supervised learning training due to their training methods and structural characteristics. To perform supervised learning training on non-geometric error compensation of rock drilling robots, various features are extracted according to different input feature vectors. The feature types are divided into three types:

(1) **Feature of the joint:** The value of each joint sensor is applied as a feature vector.
(2) **Feature of positive kinematics model:** The positive kinematics of the robotic manipulator can project the posture of each joint from the sensor to the Cartesian three-dimensional coordinate, including the motion state, spatial changes, and transformations of the non-geometric errors.
(3) **Feature of control system:** The feature of the control system refers to the signal to control the electro-hydraulic proportional valve, and the direction of joint, movement speed, and stroke distance. The hydraulic control is affected by the temperature, pressure, external load, and external environment of the hydraulic system.

### 3.4. RBFN

RBFNN is a three-layer neural network that can quickly approximate the nonlinear function to overcome local minimums. There are three layers in RBFN, namely including an input layer, a hidden layer, and an output layer, as shown in Figure 3.

The relationship of the RBFN from vector input to output is described as:


(12) $$\begin{array}{l} y_{i}=f_{i}\left(x\right)=\overset{m}{\underset{k=1}{\sum }}\omega_{ik}\phi_{k}\left(x,c_{k}\right)=\overset{m}{\underset{k=1}{\sum }}\omega_{ik}\phi_{k}\left(\theta ||x-c_{k}||\right) \end{array}$$

where *x* is the input vector, ω is the output layer connection weight, ϕ is the hidden layer basis function, and *c*_*k*_ is the basis function center of the *k*th neuron node

The radial basis kernel function is defined as Gaussian radial basis function:


(13) $$\begin{array}{l} \phi_{k}\left(\left||\theta \left(x-c_{k}\right)|\right|\right)=\exp\left[\frac{{\left(x-c_{k}\right)}^{T}\cdot \theta \cdot \left(x-c_{k}\right)}{2\sigma_{k}^{2}}\right] \end{array}$$

where σ_*k*_ is the expansion width of the radial basis function of the first neuron in the hidden layer.

Features are composed of the joint sensor value and the three-dimensional coordinates calculated through positive kinematics. In this article, the input of the RBFN model is normalized “joint sensor value (9-dimensional)” and “positive kinematics model parameters (27-dimensional)” which is 36-dimensional in total, and the output is the predicted three-dimensional coordinates. When *k* = 100 and σ = 0.05, it is validated that the model shows the best result through experiments.

### 3.5. LightGBM

LightGBM is a fast, distributed, and high-performing framework based on gradient boosting decision tree (GBDT), which has wide applications in for decades for due to its superior predictive performance. Combining gradient boosting and decision tree, lightGBM has a strong expressive ability, good training effect, and can effectively prevent overfitting by controlling the growth of the tree. It can solve continuous and discrete values flexibly and is robust to outliers. The process is mainly composed of three steps, outlined as below:

**Step 1** The training data set is given as: *P* = {(*x*_1_, *y*_1_), …, (*x*_2_, *y*_2_)}, let the loss function be *L*(*y*, γ), the learner is initialized:


(14) $$\begin{array}{l} f_{0}\left(x\right)=\arg{min}_{\gamma }\overset{N}{\underset{i-1}{\sum }}L\left(y_{i},\gamma \right) \end{array}$$

**Step 2** M base learners are iteratively generated

The negative gradient of each samples *i* = 1, 2, …, *N* is calculated as:


(15) $$r_{mi}=-{\left[\frac{\partial L(y_{i},f(x_{i})}{\partial f(x_{i})}\right]}_{f\left(x\right)=f_{m-1}(x)}$$

(*x*_*i*_, *r*_*mi*_)(*i* = 1, 2, …, *N*) is utilized as the training data for the next regression tree. The leaf node area *R*_(*jm*)_ of the m-th tree *f*_*m*_(*x*), *j* = 1, 2, …, *J*,*J* is the number of leaf nodes of the regression tree. The best fit is calculated as


(16) $$\begin{array}{l} \gamma_{jm}=\arg min\underset{x_{i\epsilon }R_{jm}}{\sum }L\left(y_{i},f_{m-1}\left(x_{i}\right)+\gamma \right) \end{array}$$

**Step 3** The strong learner is generated as:


(17) $$\begin{array}{l} {\;f\left(x\right)=f}_{M}\left(x\right)=f_{0}\left(x\right)+\overset{M}{\underset{m=1}{\sum }}\overset{J}{\underset{j=1}{\sum }}\gamma_{jm}I\left(x\epsilon R_{jm}\right) \end{array}$$

The endpoint error compensation is used as the training label for supervised learning; its training features have a total of 66 dimensions, which are “feature of the joint (9 dimensions)” + “feature of positive kinematics model (27 dimensions)” + “feature of control system (30 dimensions).” K-fold cross-validation method is applied. The test data *D* is divided into k parts in equal proportions. One of the parts is applied as the verification set. The other *K*−1 parts of data are used as the training data. After performing K times of training, the average result of the K training experiments is used as the final result. When the model stability is low, increasing the value of K can achieve better results.

## 4. Experimental Validation and Results

### 4.1. Experimental Set-Up

In order to achieve accurate position compensation for the rock drilling robot, the error compensation experiment is performed. In this experiment, the SWDT82 rock drilling robot developed by Sunward Intelligent Co., Ltd, as shown in Figure 5, has a maximum manipulator length of 12.8 m and a wingspan of 5.2 m. The maximum coverage section width of the dual-manipulator rock drilling robot is 17.65 m, the maximum coverage section height is 13.38 m, and the maximum invert depth is 3.31 m.

The rock drilling robot is equipped with a three length transducer and seven angle sensors as shown in Figure 6. The angle sensors are the rotary encoder of POSITAL's model IXARC, and its theoretical measurement error is 0.1°; the length transducer is TRANSTRONIC's model 022441, and the theoretical error of measurement linearity of this sensor is 0.1% mm. The observation instrument in the experiment is a Topcon laser total station, which has functions such as motor tracking, automatic sighting, and Bluetooth transmission.

### 4.2. Experimental Results

**Figure 7:**
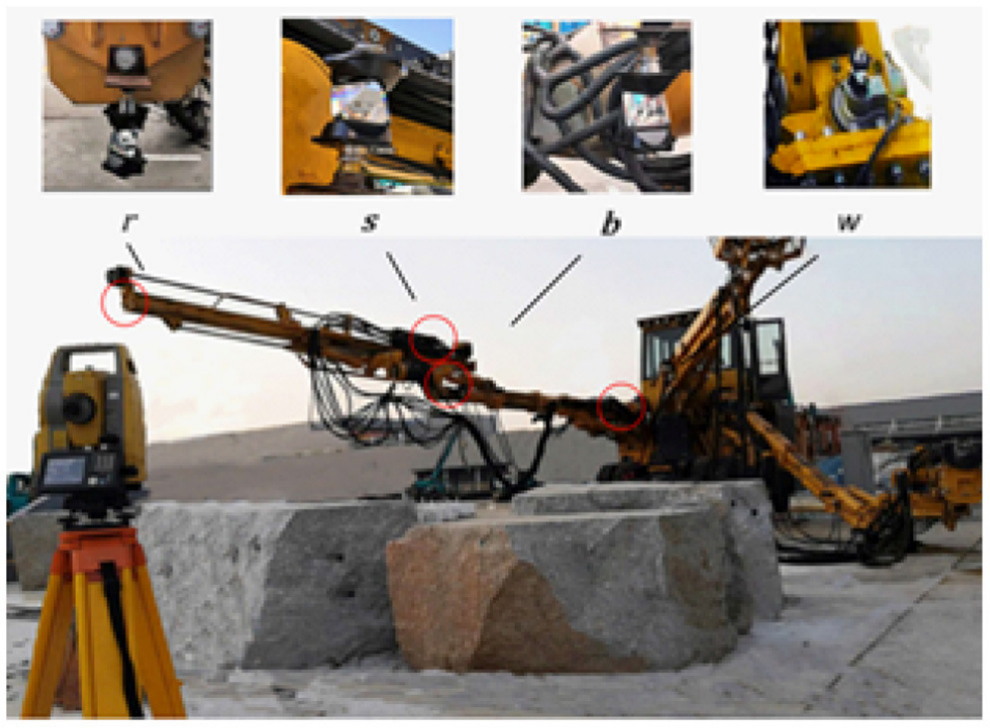
Experiment of the rock drilling robot.

**Figure 8:**
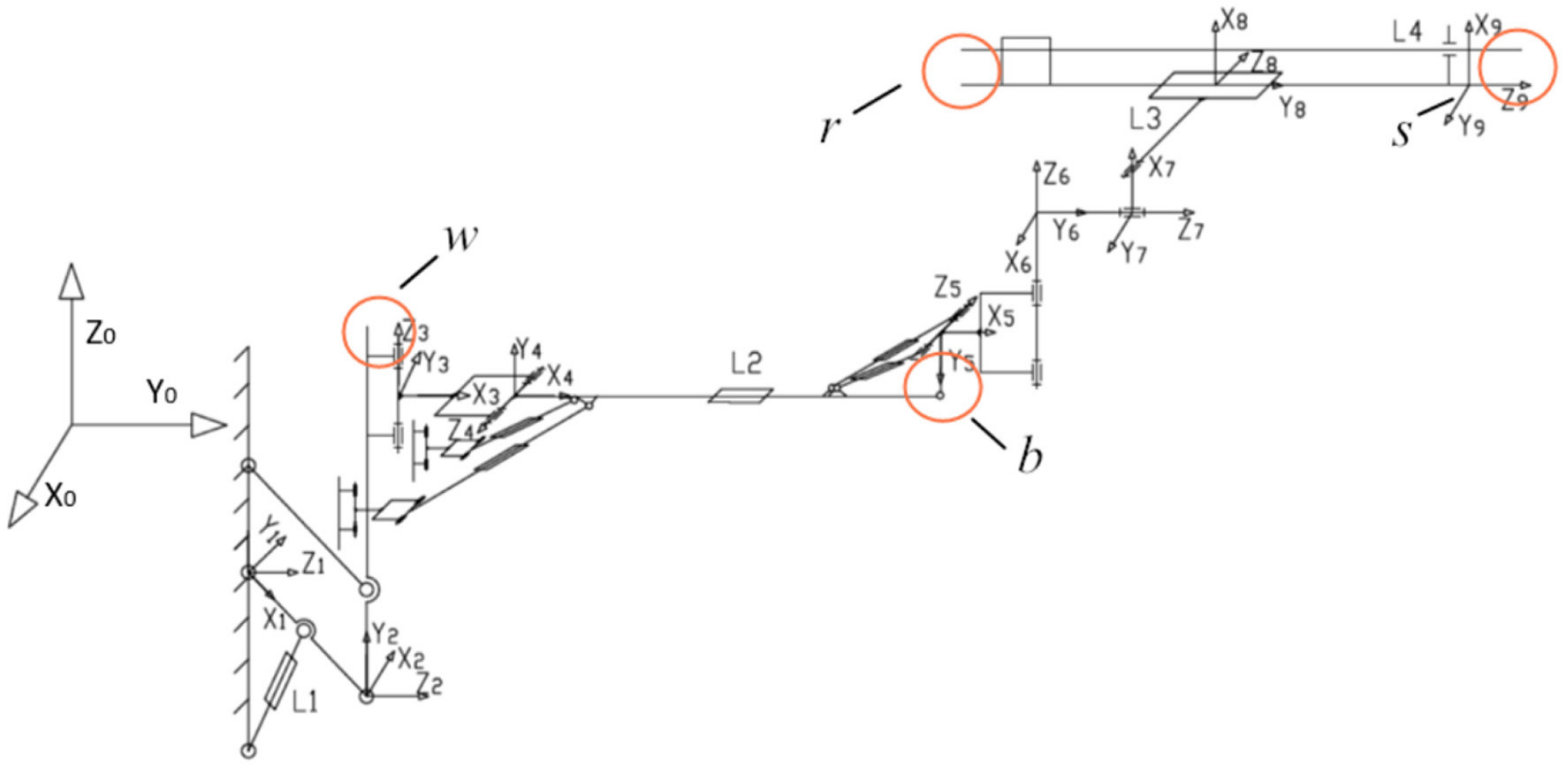
Schematic diagram of observation point.

To compensate for the geometric error of the rock drilling robot, the motion of a single joint is carried out. The position and posture of the rock drilling robot are collected through Topcon total station transmitting to the master computer using Bluetooth as shown in Figure 7. The data of the angle sensors and length transducers are transmitted to the master computer *w*, *b*, *r*, and *s* corresponding to the joint 3, the joint 5, the front of the compensation cylinder 9, and the end position of the compensation cylinder 10, as shown in Figure 8. Moreover, the end posture of the rock drilling manipulator is determined by the position of observation point *r* and *s*.

So as to identify the geometric error of the manipulator, single-joint motion of the manipulator is carried out. After substituting each observation point, obtained from sensors, into the forward kinematics model for calculation, the absolute errors can be obtained. The absolute average error of each observation point is $\bar{e_{r0}}$: $\bar{e_{s0}}$: $\bar{e_{b0}}$: $\bar{e_{w0}}$ = 323.59: 477.53: 895.52: 930.88 (mm).

Afterwards, the multi-joint motion experiment is carried out. Trajectory data of the observation points of rock drilling robot motion *r*, *s*, *b*, *w* is applied for error analysis. In the multi-joint movement, the absolute average error of each observation point data set $\bar{e_{r0}}$: $\bar{e_{s0}}$: $\bar{e_{b0}}$: $\bar{e_{w0}}$ = 355.61: 783.17: 1085.32: 1288.916 (mm).

In order to identify the forward kinematics of the manipulator, the differential evolution method is applied to compensate for the geometric error. This experiment not only explores the error compensation effect of the error model method but also explores the influence of the amount of data on the convergence of the parameter identification of the error model method. Due to the high-dimension of the model, the population of the differential evolution algorithm is set to 30,000. To avoid premature convergence of parameter optimization, the differential cross recombination probability *C*_*r*_ is set to 1, the scaling factor *F* = 0.5, and the number of evolutions is set to 400. To improve the performance of the model, the data set is shuffled and substituted into the model multiple times to obtain the optimal solution. To optimize the error model, the joint constraints are increased. Set the end trajectory of each observation point as $L_{w}^{\prime }$, $L_{b}^{\prime }$, $L_{s}^{\prime }$, $L_{r}^{\prime }$, the trajectory observation point set is *P*′ and the model prediction point set are (*X*′, *Y*′, *Z*′, Then the position error of each observation position are $e_{w}^{d},e_{b}^{d},e_{s}^{d},e_{r}^{d}$. The average absolute error *F* of the optimal error model with ideal multi-joint constraints at each observation point is expressed as:


(18) $$\{\begin{array}{l} F_{w}=\min\left(e_{w}^{d^{2}}\right)\\ F_{b}=\min\left(e_{b}^{d^{2}}\right)\\ F_{S}=\min\left(e_{S}^{d^{2}}\right)\\ F_{r}=\min\left(e_{r}^{d^{2}}\right) \end{array}$$

The solutions of *F*_*w*_, *F*_*b*_, *F*_*s*_, *and F*_*r*_ are difficult to satisfy simultaneously, and the reduction of the error of some joint clusters requires the degradation of the error fitting effect of the other joint clusters, resulting in the Pareto solution. And new observation errors are introduced by adding trajectory constraints, which reduce the overall accuracy of the model. In order to solve the above problems, an equal amount of data in the model calculation are applied for each joint. Moreover, a weight coefficient is assigned to each joint, and the functions are obtained by linearly combining the multi-objective functions:


(19) $$\begin{array}{l} F\left(\Delta \alpha ,\Delta a,\Delta d,\Delta \theta ,\Delta \beta \right)=\frac{\mu_{r}e_{r}^{d^{2}}}{n_{r}}+\frac{\mu_{s}e_{s}^{d^{2}}}{n_{s}}+\frac{\mu_{b}e_{b}^{d^{2}}}{n_{b}}+\frac{\mu_{w}e_{w}^{d^{2}}}{n_{w}} \end{array}$$

where μ_*r*_, μ_*s*_, μ_*b*_, *and μ*_*w*_ are the weight coefficients of the multi-objective error constraint, and*n*_*r*_, *n*_*s*_, *n*_*b*_, *and n*_*w*_ are the numbers of each data set. The parameters of forward kinematics of the optimal method are obtained shown in as Table 2.

**Table 2:** Parameters of positive kinematics compensation.

***i***	**Δ*α*_*i*−1_/(°)**	**Δ*a*_*i*−1_/mm**	**Δ*d*_*i*−1_/mm**	**Δ*θ*_*i*−1_/(°)**	**Δβ*i*−1/(°)**
1	3.5932	266.49	−37.92	3.9988	0
2	0.3292	−91.55	85.62	2.3400	5.3136
3	5.9856	90.88	96.04	2.9599	0
4	−5.7576	95.11	369.38	5.1696	−2.9444
5	−3.1405	123.58	230.58	1.1894	0
6	−0.5786	−253.95	−33.82	−0.5875	0
7	1.0460	269.17	225.12	4.6678	0
8	−1.0995	376.07	24.36	2.8406	0
9	3.5932	266.49	−37.92	3.9988	0

The average absolute error can be calculated from the compensated positive kinematics. A parallel differential evolution algorithm is applied to move a single joint as the first experiment. Then, a parallel differential evolution algorithm is applied for moving multi-joints applying as the second experiment. Ultimately, the optimal differential evolution algorithm is applied as the third experiment. Consequently, the distribution of errors is shown in Table 3.

**Table 3:** The mean absolute error of each experiment.

	**ē_*r*_ (mm)**	**ē_*s*_ (mm)**	**ē_*b*_ (mm)**	**ē_*w*_ (mm)**
Single joint	382.58	396.04	466.92	263.21
Multi joint	389.08	334.58	290.45	44.83
Optimal method	306.82	320.12	298.69	70.37

Applying the optimal method of parallel differential evolution, the errors of the end effectors are compensated, and the best performance is achieved compared to the parallel differential evolution algorithm.

**Figure 9:**
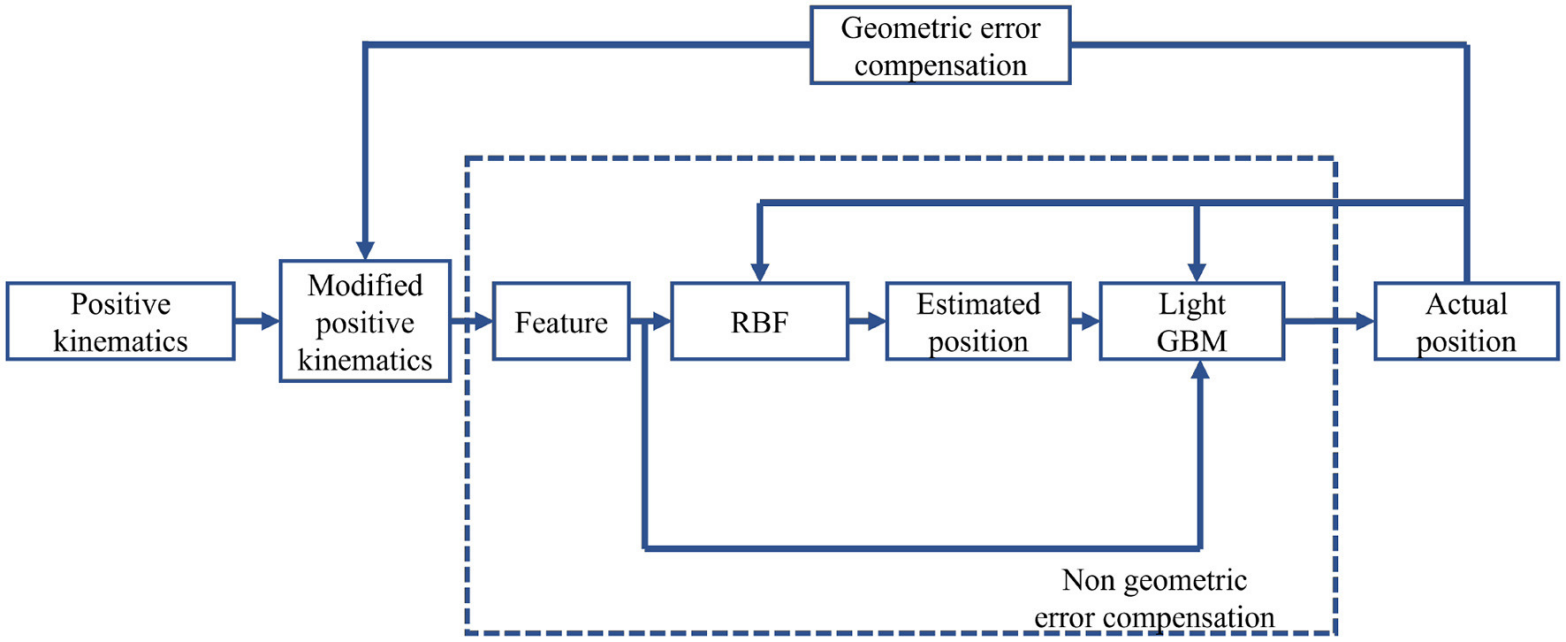
Control flowchart of the rock drilling robot's error compensation.

**Figure 10:**
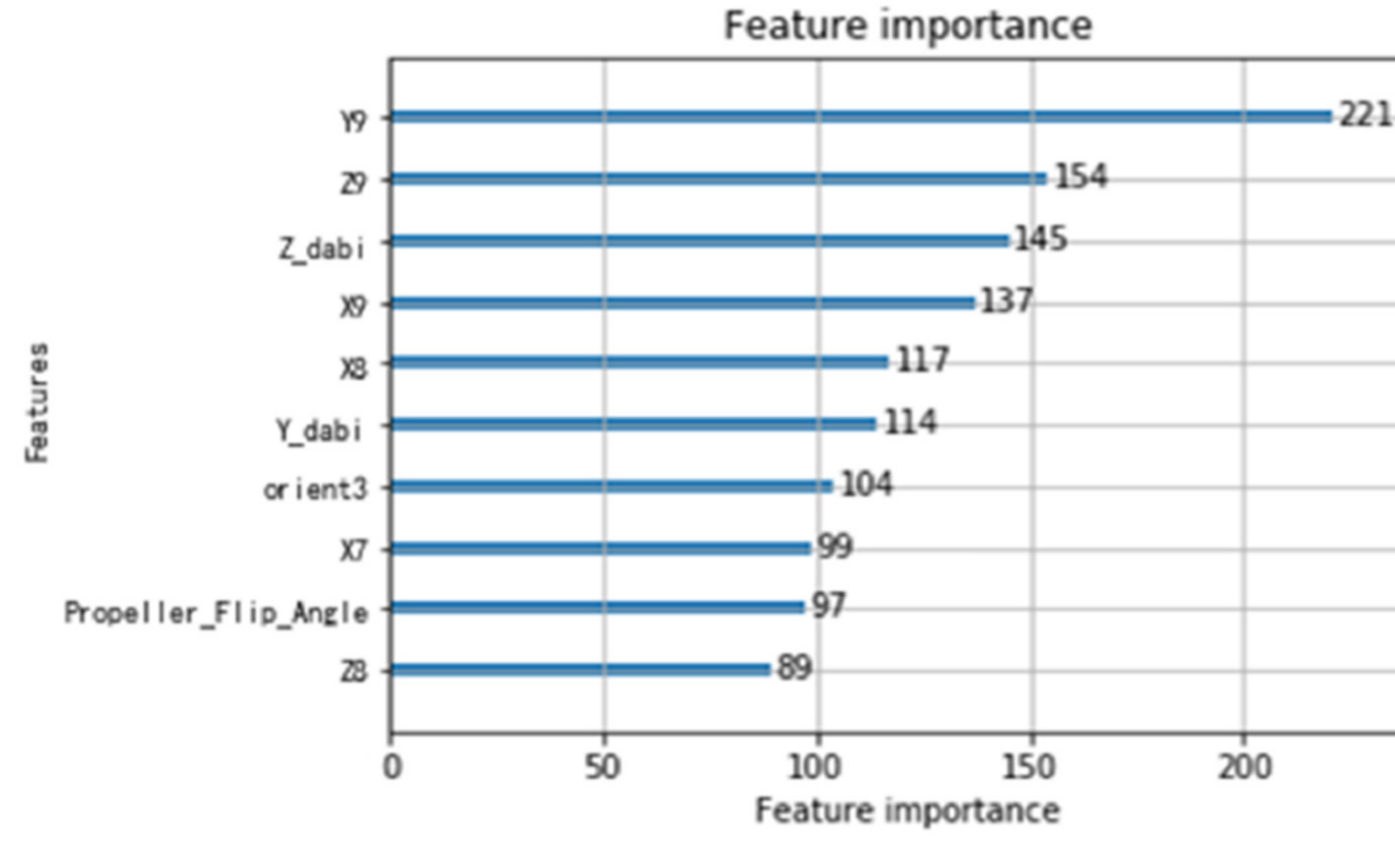
Top 10 features of lightGBM.

The overall control flowchart is shown in Figure 9. Firstly, the modified positive kinematics is obtained through an optimal parallel differential evolution algorithm to compensate for the geometric error. Afterward, the features are applied to train RBFN. The estimated position and features are combined to train light GBM. The non-geometric errors are compensated through RBFN and light GBM. The top 10 features are shown in Figure 10. *X*_*i*, *Y*_*i, Z*_*i* (i=1, 2..) are the Cartesian coordinates of the joint i, *orient*3 is the control current of the hydraulic valve, *Propeller*_*Flip*_*Angle* is the sensor of the flip joint, *Z*_*dabi, Y*_*dabi* denote the Z coordinate and Y coordinate of the big manipulator separately, Y coordinate. The hydraulic valve current control ranks 7th in feature importance as a system feature, indicating that the system feature has an important contribution for the model.

**Figure 11:**
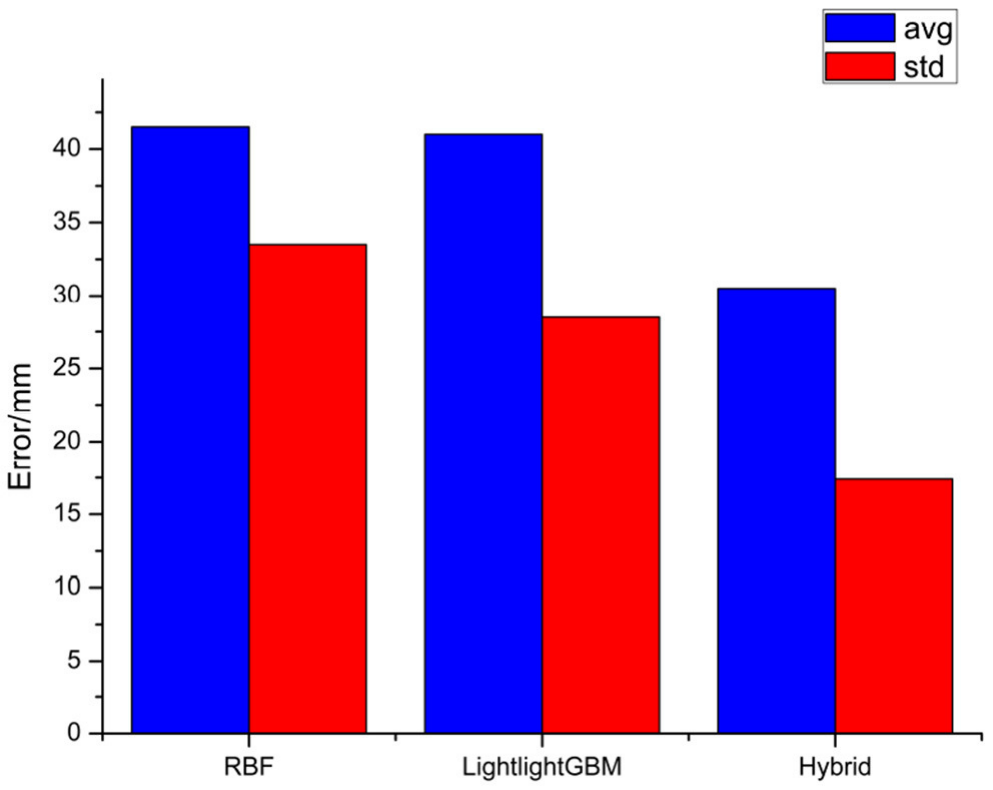
The absolute error of the end point.

The absolute error of the endpoint is obtained for multiple experiments in Figure 11. All of the experiments are carried out by applying the same positive kinematics from the optimal parallel differential evolution. The average error of the RBFN is 41.5 mm, while the standard error of the RBFN is 33.5 mm. Moreover, the average error of the lightGBM is 41.0 mm, while the standard error of the RBFN is 28.5 mm. Moreover, the average error of the hybrid method is 30.5 mm, while the standard error of the hybrid method is 17.4 mm. It can be concluded that the hybrid method can achieve a good performance.

**Figure 12:**
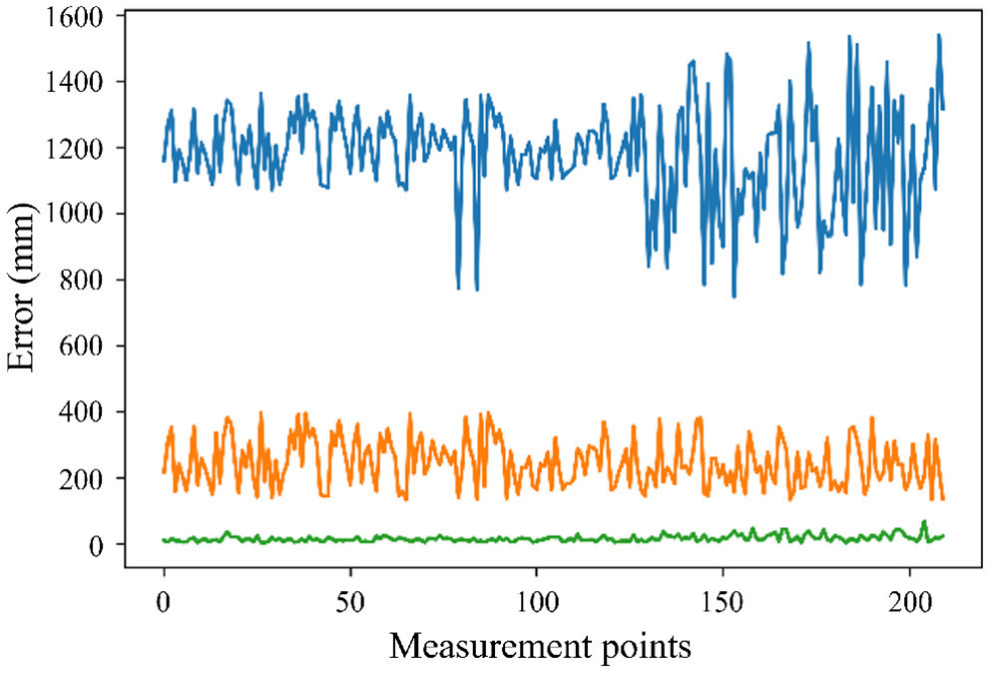
The absolute error before compensation (blue line), the absolute error applying geometric compensation (yellow line), the absolute error applying geometric compensation and non-geometric compensation (green line).

A validation experiment is carried out to verify the effectiveness of the proposed method as shown in Figure 12. Two hundred points collected along the trajectory are analyzed in two error compensation methods. The blue line is the absolute error before compensation. The yellow line is the absolute error applying the parallel difference algorithm for geometric compensation. The green line is the absolute error applying RBFN combined with light GBM for geometric compensation and non-geometric compensation. It can be concluded that the error of the rock drilling robot is compensated effectively by applying the proposed method, which meets the practical application needs of rock drilling robots.

## 5. Discussion and Summary

Geometric error compensation and non-geometric error compensation methods are combined in this article. Firstly, the positive kinematics model of the rock drilling robot is established by the MDH method. Then, a parallel differential evolution algorithm is designed to identify the forward kinematic parameters to compensate for the geometric error. Single joint and multi-joint experiments are carried out to analyze the error. By analyzing non-geometric error through experimental trajectories, non-geometric error compensation based on RBF neural network and lightGBM is proposed. Moreover, feature engineering for the rock drilling robot is analyzed based on the robotic kinematics model to achieve the optimization of the non-geometric error compensation model. Finally, the experiment is carried out to validate the performance of the proposed method, which meets the precision requirements of rock drilling robots. This article has shown that the addition of positive kinematic parameters and control signal parameters as feature engineering can greatly improve the learning ability of machine learning. Therefore, features such as speed, oil pressure, and artificial deflection calculation can be considered in the research of machine learning to further improve the learning ability and generalization of the model. And the deflection deformation of the manipulators can be modeled and analyzed in the field of mechanical materials to enhance positional accuracy.

## Data Availability Statement

The raw data supporting the conclusions of this article will be made available by the authors, without undue reservation.

## Author Contributions

XZ contributed to the development of the simulation engine of the platform under the guidance of JH, YZ, and GB. JD and PL contributed to experimental acquisition and algorithm verification. XZ and WB contributed to analysis of the data from simulation and physical experiments. All authors contributed to writing, reviewing, and proofreading the manuscript.

## Funding

This research was supported by the National Natural Science Foundation of China (52105127) and the Natural Science Foundation of Zhejiang Province, PRC (LGG22E050025).

## Conflict of Interest

WB was employed by Zhejiang Jinbang Sports Equipment Co. Ltd. PL and YZ were employed by Sunward Intelligent Equipment Company, Ltd. The remaining authors declare that the research was conducted in the absence of any commercial or financial relationships that could be construed as a potential conflict of interest.

## Publisher's Note

All claims expressed in this article are solely those of the authors and do not necessarily represent those of their affiliated organizations, or those of the publisher, the editors and the reviewers. Any product that may be evaluated in this article, or claim that may be made by its manufacturer, is not guaranteed or endorsed by the publisher.
